# The Cognitive Aspect of Hope in the Semantic Space of Male Patients Dying of Cancer

**DOI:** 10.3390/ijerph20021094

**Published:** 2023-01-08

**Authors:** Bożena Baczewska, Krystyna Wojciechowska, Beata Antoszewska, Maria Malm, Krzysztof Leśniewski

**Affiliations:** 1 Department of Internal Medicine and Internal Medicine in Nursing, Faculty of Health Sciences, Medical University of Lublin, Chodźki 7, 20-093 Lublin, Poland; 2 Department of Strategy and Business Planning, Lublin University of Technology, Nadbystrzycka 38 Street, 20-618 Lublin, Poland; 3 Department of Special Needs Pedagogy and Resocialization, Faculty of Social Sciences, The University of Warmia and Mazury in Olsztyn, Żołnierska 14, 10-561 Olsztyn, Poland; 4 Department of Medical Informatics and Statistics with e-Health Lab, Medical University of Lublin, Jaczewskiego 4, 20-090 Lublin, Poland; 5 Department of Orthodox Theology, Faculty of Theology, The John Paul II Catholic University of Lublin, Al. Racławickie 14, 20-950 Lublin, Poland

**Keywords:** hope, terminal stage of cancer, semantic differential, palliative and hospice care

## Abstract

The aim of this study is to characterize the cognitive aspect of the semantic space of hope in patients in the terminal stage of cancer. This was confirmed in the research on hope by C. R. Snyder and B. Schrank. Hope is of great importance in all the great world religions and belief systems, both as regards a personal God or impersonal deities. Hoping is a human capacity with varying affective, cognitive and behavioral dimensions. Psychological, pedagogical (particularly in the framework of special needs pedagogy and thanatological pedagogy) and theological reflection on hope can provide support for dying people. In order to conduct the research, the semantic differential research method was selected. The research technique employed was a therapeutic conversation, and the research tool was the B.L. Block’s DSN-3 test. The DSN-3 test allows one to assess hope in the semantic space in three aspects: cognitive, emotional and functional. For the purposes of this study, only the cognitive aspect was taken into account. The study was begun on 1 April 2010 and ended in the last days of December 2020. It included 110 male patients in the terminal stage of cancer. The youngest respondent was 19 years old and the oldest was 94 years old. The surveyed men most often perceived hope in the semantic space in the cognitive aspect as more true, wise, meaningful and real than false, stupid, meaningless and deceptive. Their attitude to hope was, therefore, more affirmative than negative. The research did not reveal the importance of the age of the respondents on the degree of affirmation/negation of hope in the cognitive aspect in the semantic space; however, men in the period of late maturity and professional activity expressed the lowest level of the affirmation of hope. It is worthwhile to conduct further research concerning hope in other aspects (especially emotional and functional) in the semantic space in order to use the obtained results to consider what to take into account when providing patients in the terminal stage of cancer with better personalized holistic care than before.

## 1. Introduction

Human existence depends not only on the relationship with the present or remembering what happened in the past but also on anticipating what will happen in the future, both having a positive and negative impact. “Hope” (Gr. *elpis*), in its essence, is a kind of subjective projection of the future but only in relation to what is expected to be good. It should be stressed that hope is a reality that is difficult to specify or define precisely. For the ancient Greeks, hope served as comfort in states of misfortune, distress, anguish or affliction [1]. Over the centuries, hope has been very important in the reflection on human existence, both in broadly understood science and everyday life. Nowadays, there are at least several dozen different definitions of hope, depending on the adopted initial assumptions in a given field of science and existential experience.

Depending on the concept, there are various components of hope. For example, in 2008, Schrank, Stanghellini and Slade [2] analyzed and synthesized scientific publications devoted to hope. They concluded that there were 49 definitions of the concept of hope and proposed their own definition of hope, describing it as a state primarily directed towards the future in such a way as to achieve a goal or relationship of spiritual importance for a person. Dufault and Martocchio [3] emphasized that hope is a multidimensional life force that includes expectations/beliefs that a person will gain the good in the future that one is waiting for and that is important to that one, as well as that obtaining this good is achievable. According to these authors, hope should be understood as a set of many different thoughts, feelings and actions that are subject to change. As indicated by Morse et al. [4,5], hope is a response to a threat, as well as an activity that overcomes helplessness and a factor that reduces suffering. Hope, in Krafft et al.’s conception [6], is defined as the feeling of having a deep trust in the positive effects of difficult situations, especially those that are beyond direct human control. Regarding hope, this author combines two points of view: cognitive/individualistic with transcendent/emotional.

Hope is one of the most important predictors and motivators for human action. Every human person experiences hope at a different level of intensity, whereas this intensity is dynamic. Paulo Freire underlines that “hope, as an ontological need, demands an anchoring in practice” [7]. A threat to human existence is a crisis of hope, usually described as “hopelessness”, which can lead to a state of despair [8,9,10]. Thanks to hope, a person is able not only to go beyond the limitations one experiences in one’s present life but also to become used to the fear of impending death, especially when experiencing the terminal stage of a disease [11]. Since the earliest times, hope has been associated with faith (Gr. *pistis*) and love (Gr. *eros*) and treated as one of the most important aspects of a person’s spiritual attitude, as well as a means of conditioning an authentic life. Fundamentally, hope is the expectation of good, coupled with trust, as well as yearning and strength to counter existential fears. Hence, hope is not a delusion or a dream but a persistent expectation of experiencing good in the future and, sometimes, even absolute happiness.

Hope is of great importance in all the great world religions and belief systems, both as regards a personal God and impersonal deities. In Christianity, which significantly shaped the humanistic foundations of European civilization, hope is related to Jesus Christ and was essentially an expectation of an eternal life beyond physical death. Hope, in such an approach, is a great vital force for humans, enabling them to maintain a sense of the meaning and purpose of life, especially in the state of overwhelming suffering. For many Christians, hope in combination with faith in God has been equated with trust that everything will be fine in the end [12]. This understanding of hope resulted from the belief that biological death is not the end of a person’s life and the disintegration of one’s personal identity. From linking hope with faith in a personal God, Christians gain the vital energy to fight incurable diseases and to overcome the paralyzing fear of death more effectively.

Hope, in connection with faith and love, does not just depend on the belief in the existence of God or deities but is one of the features of a person’s identity. Each person, regardless of their religious beliefs, negation or acceptance of further existence after death, in making choices, is guided by hope or its opposite: hopelessness/despair [13]. Especially in borderline situations, such as the final stage of neoplastic disease, hope (or its opposite—hopelessness/despair) directly and indirectly affects the condition of the patient. Depending on whether and to what extent terminally ill patients choose the attitude of hope as a reference point for determining the meaning and purpose of their life, their physical condition and their spiritual and mental state, as well as the quality of their relationships with other people (social condition) improve or deteriorate [14]. Those dying of cancer find themselves in a situation that forces them to choose whether to opt for hope (even against a diagnosis of ill-health) or hopelessness/despair [15,16].

From a medical point of view, research (with the use of psychological methods) on defining hope in patients in the terminal stage of neoplastic disease in the semantic space in terms of cognitive, emotional and functional aspects is very helpful [17]. Although this type of research does not provide full cognitive insight into the richness of patients’ spiritual and mental lives, it must be stressed that these patients recognize the enormous antientropic and pro-existential potential of hope as a life force in their ultimately doomed fight against cancer [18]. In the cognitive aspect, determining hope by the pairs of semantically antithetical adjectives makes it possible to examine the beliefs of terminally ill patients within their subjectively experienced rationality. With regard to the emotional aspect, it should be noted that it is difficult to make an in-depth analysis of the richness of a person’s spiritual and mental life. Nevertheless, referencing the pairs of adjectives, which provide a description of hope in the emotional and experiential space, also makes it possible to draw conclusions about one’s spiritual life, even without taking into account religious beliefs. Investigating the functional aspect, which is so important today from a social point of view, helps to perceive hope as a reality that stands in opposition to hopelessness/despair in the experience of terminal cancer patients.

In 1999, Nekolaichuk et al. studied hope using the semantic differential technique in Alberta, Canada [19]. So far, no similar studies have been conducted on the Polish population.

This article focuses only on the presentation and evaluation of the results of research relating to the cognitive aspect of hope in the semantic space of patients in the terminal stage of cancer. The emotional and functional aspects of hope in the semantic space will be the subject of further studies. 

### 1.1. Essential Concepts of Cognitive Semantic Space

The presentation of the results of research on the cognitive aspect of hope in the semantic space of male patients dying of cancer requires defining what semantic space is and indicating the designations of opposing adjectives by which the surveyed men described their hope in this aspect. “Semantic space” is the spatial structure of the representation of concepts relating to objects from the social environment. It is one of the regulators of human behavior, as there may be different distances between particular representations. The representations of individual pairs of semantically contradictory adjectives may be spatially closer to each other or farther apart. In this study, a seven-point scale was used for concepts that were semantically contradictory. Moreover, a numerical value was assigned to perform statistical calculations.

“True” and “false” are two basic logical values. As commonly understood, based on the classical philosophical definition, truth is a property of human judgments that consists in their compliance with the actual state of affairs they concern. Nowadays, there is a noticeable tendency to understand truth as an universally binding cognitive value. In this approach, judgment in the logical sense is the primary carrier of truth. The opposite of truth is falsehood—wherein the content of the expressed judgment or sentence is inconsistent with what it refers to. Within the framework of traditional logic, falsehood is the statement of something that did not or will not actually exist, or the denial of something that has or may have happened. With regard to hope, truth and falsehood can only be considered in the cognitive aspect as the expectation of both a positive state of affairs and the uncertainty or doubt that this will happen. The subjective expectation regarding a high probability that a given expectation or desire of a person will be fulfilled is expressed by the statement that hope is true. On the other hand, great uncertainty or the feeling that there is no chance for the fulfillment of a given expectation or desire of a person is expressed by the statement that hope is false.

“Wisdom” is one of the most fundamental concepts in human life. In essence, however, wisdom is very difficult to define, because its designate depends on various preliminary assumptions that relate to philosophical, religious, social and cultural premises. Nowadays, wisdom is usually treated in relativist and rationalistic terms, as a kind of an optimal compromise between the ideal and reality. Commonly, wisdom is understood as acquired knowledge, thanks to which one has the ability to assess matters with the greatest objectivity, as well as to make thoughtful and responsible choices aimed at the broadly understood good, both in relation to oneself and other people. The opposite of wisdom is stupidity, which is equated with a lack of knowledge, intelligence or reason, as well as with ignorance or disregard for generally accepted social norms. Stupidity is present in all areas of life and in every civilization. It is manifested by mental sluggishness in speech and action, as well as an inability to associate facts, determine cause-and-effect relationships between facts and predict the effects of various types of interactions and actions. When it comes to hope, wisdom and stupidity can be viewed in the cognitive aspect. “Wise” hope in a patient in the terminal stage of neoplastic disease is characterized by a sober assessment of reality based on one’s medical knowledge and understanding of one’s situation in order to focus as much as possible on the good that is the value of one’s life. In opposition to it, “foolish” hope is an illusory vision of recovery and images focused on recovery and the realization of one’s life plans.

“Meaning” is a multifaceted term. Usually, meaning is either identified with the positive (i.e., good in effect) goal of an action (intentional sense), or it is treated as the essence of the meaning of a given concept in relation to experience (cognitive sense). Meaning, without relation to experience, means focusing on various types of imaginative thoughts, as a result of which a person discovers readiness to act. In the common understanding, the opposition “meaningful–meaningless” is used to express the purposeful and justified or pointless and unjustified orientation of the human will. With regard to hope, the opposition “meaningfulness and meaninglessness” can be studied in the cognitive sphere as a kind of reflection on the purposefulness of expecting something that is beneficial and good for its effects, or supposing that there are no grounds for realizing the desired good.

“Reality” is usually conceived of as being consistent with the facts or the possibility of making something real. In the common understanding, reality is something real that actually exists. In opposition to reality, there is “deception”, that is, something that is a delusion, a ridiculous dream, a hallucination or an illusion and, therefore, a false image—a product of human imagination. With regard to hope, the opposition “real–deceptive” has a cognitive meaning, because it concerns the process of thinking aimed at anticipating what is to happen in the near or distant future. Someone who has “real” hope is someone who has sufficient premises to expect some good in the future, whereas a person has “deceptive” hope when one completely unreasonably and based on illusions expects that one’s desire will come true. Deceptive hope is rooted in a false and naive faith, an unrealistic dream or a mistaken feeling that is the result of distorted sense impressions. The conducted research revealed a very different perception of “real hope–deceptive hope” in the semantic space by patients in the terminal stage of neoplastic disease.

### 1.2. Objectives of the Study

The aim of this study was to characterize the cognitive aspect of the semantic space of hope in patients in the terminal stage of cancer. This aspect is crucial, since it dominates the structure of hope as it contains beliefs about planned future achievements, as well as images, thoughts and associations. This was confirmed in the research on hope by C. R. Snyder and B. Schrank [1,20]. Focusing on the future, cognitively defined, is of particular importance for people in the terminal stage of neoplastic disease. In this study, we considered the attitude of terminally ill male patients to four pairs of adjectives characterizing hope and hopelessness/despair in the cognitive aspect (“false–true”, “stupid–wise”, “meaningless–meaningful” and “deceptive–real”). The main research problem was the search for an answer to the question: How do male patients in the terminal stage of neoplastic disease characterize their hope in the semantic space in the cognitive aspect? We also considered whether the age of the respondents influenced the degree of affirmation/negation of hope in the cognitive aspect of the semantic space. The attitude of these patients to the four pairs of adjectives indicated above was the basis for determining the degree of affirmation/negation of hope in the cognitive aspect in confrontation with the fact that their own death is imminent and for drawing practical conclusions as to providing them with the best personalized holistic medical care.

## 2. Materials and Methods

In order to conduct the research, the Osgood semantic differential research tool was used [21]. The semantic differential is applied to measure the meaning of concepts. Thanks to this, the subject defines the word using semantically opposite adjectives placed at a certain distance opposite to each other. The task of the examined person is to mark the selected point between the pairs of adjectives in such a manner as to be closer to that which expresses his/her own feelings, beliefs or attitude [3,22,23]. The research technique employed was a therapeutic conversation [21], and the research tool was the B. Block’s DSN-3 test. This tool allows the researcher to describe hope using connotations in three aspects: cognitive, emotional and functional—as distinguished by factor analysis. The parametric properties of this test were developed by B. Block on a sample of *n* = 306, and the validity and reliability of the test were determined as Cronbach’s alpha for the overall score of 0.91 and for individual aspects of hope (cognitive aspect, evaluative–emotional aspect and functional aspect) of 0.80–0.84.

### 2.1. DSN-3 Research Tool Design

The list of pairs of adjectives was created based on the method of “Competent Judges” (10 psychologists, including 4 doctors of psychology). The judges assessed the adjectives according to two criteria. Their common understanding (in Polish)—they come from everyday language and do not go beyond the conceptual framework. The judges defined their semantic opposition.

From the list of 87 pairs of opposite adjectives (DSN-1), 40 pairs of adjectives were selected by discriminant analysis. The DSN-2 was subjected to factor analysis, as a result of which three factors were distinguished, referring to the Osgood nomenclature: (a) truth (cognitive), (b) evaluating (evaluative-emotional) and (c) regulatory (functional). Due to the purpose of the test, for people in the terminal phase of cancer, it was shortened as much as possible. As a result, 4 pairs of adjectives were selected for each of the factors, in total, 12 pairs with the highest discriminatory power—DSN-3.

Statistical analyses were the basis for the selection of pairs of opposing adjectives. After applying a factor analysis, three factors were identified, the first of which is the cognitive factor—“truthfulness”. It turned out that it is enough to use only four pairs of opposing adjectives with the highest relevance and at the same time a very high “factor input”. The severe psychophysical condition of people for whom this test was dedicated was taken into account. Despite the small number of pairs of opposite adjectives, factor analysis is considered sufficient to describe the concept under study [24].

### 2.2. Ethical Considerations

This research was conducted in a friendly atmosphere by a person well known to the patient. For ethical reasons, the study was performed by a qualified nurse caring for this patient. The examination often turned into a therapeutic conversation. All respondents gave their consent to participate in the study and were informed about the confidentiality of their personal data. A team of researchers, led by B. Block, decided to conduct the research after obtaining a positive opinion from the Bioethics Committee at the Medical University of Lublin (opinion no. KE-0254/225/2010).

### 2.3. DSN3 Scale—Construction of the Questionnaire

The tool sheet consisted of pairs of opposing adjectives arranged as antonyms, between which seven degrees of affirmation were indicated (from “definitely negative” to “definitely positive”). The choice of the degree of a positive or negative rating (definitely negative, negative, rather negative, rather positive, positive and definitely positive) relating to a given adjective characterizes the respondent’s perception of hope in this space. Assessing hope in the semantic space in the cognitive aspect, the respondent defined it by the following pairs of adjectives: “true–false”, “wise–stupid”, “meaningful–meaningless” and “real–deceptive”. The average results were interpreted. In order to assess the perception of hope by the respondents, the following class ranges were used: 1.0–1.99—definitely negative, 2.0–2.99—negative, 3.0–3.99—rather negative, 4.0–4.99—rather positive, 5.0–5.99—positive, and 6.0–7.0—definitely positive. Both Rensis Likert [25] and Osgood et al. [24,26] applied scaling of responses to research using questionnaires. These researchers assumed that for a large number of subjects, equal distances on the scale between each of the points in relation to semantically opposite pairs of adjectives can be considered.

### 2.4. Conducting a Study among Men Dying of Cancer

The study was conducted from 1 April 2010 to the end of December 2020 and included 110 male patients selected from 306 people in the terminal stage of cancer. The reason for this choice was the desire to obtain information about the hope of men in the terminal phase of cancer. Numerous studies show that men cope worse in extreme situations and more easily than women fall into helplessness, helplessness and apathy or aggression or self-aggression, which sometimes in this extreme situation of enormous suffering with no chance of healing makes them think about the desire to end their lives as soon as possible (euthanasia) [27,28]. Furthermore, research suggests that men are much more rational in their approach to life than women, who are more emotional by nature [29].

The selection of the sample for the study was purposeful: those with a diagnosed terminal phase of cancer. People with impaired consciousness and lack of logical thinking were not included in the study. The subjects met the criteria of communicativeness and gave their informed consent to the study before its commencement. The subjects answered the questions from the questionnaire anonymously. Many (17) centers participated in the study. These were palliative medicine wards, stationary hospices and home hospices.

The research was carried out in a friendly and supportive atmosphere. In the event of difficulties, patients received support and explanation. The answer sheet had detailed instructions on how to mark the correct answer. This manual described the construction of the questionnaire and how to mark answers on the scale between opposing adjectives. There was also an example supplied illustrating how to mark the answer, namely: “Put an X closer to the adjective that is consistent with how you perceive it, i.e., what you feel, think and believe. There are no right or wrong answers here”. The adjectives were taken from everyday language, commonly understood by the average person. They were, therefore, fully understandable and did not require further explanation. The respondents were very willing to give their answers. They perceived the survey as easy, fully understandable and pleasant to fill out.

For the purposes of this study, a group of male patients was separated from the entire group of respondents. In total, 110 men were qualified for the study, their average age being 60.15 ± 13.53. The youngest respondent was 19 years old, while the oldest was 94 years old. Most men were under the care of a home hospice (*n* = 53, 48.18%) or stationary hospice (*n* = 51, 46.36%) and only some stayed in palliative medicine wards (*n* = 6, 5.46%). The characteristics of the study group are presented in Table 1.

In addition to examining the semantic differential of hope in the study group, researchers also wondered whether there were differences in the perception of hope in the semantic space in the cognitive area due to the respondents belonging to different age groups yet at the same stage of cancer (terminal phase). The surveyed men were thus classified into four age groups, taking into account, first of all, professional activity throughout their lifespan. Typically, the first of these four age groups (18–35—youth and the beginning of professional activity) included people who had obtained vocational education, found a permanent job and consciously developed their competences in the scope of undertaken professional activities. The second age group (36–50 years—early maturity and the highest professional activity) comprised people who were at the peak of their professional potential, were promoted and had achieved various types of success and were usually in good to very good physical condition. The third age group (51–65 years old—late maturity and the period of late professional activity) included people who had already gained a lot of professional experience, had numerous achievements, sometimes had a significant social status and at the same time were already to some extent tired of life struggles and were starting to prepare for retirement. The fourth age group included people (over 65—retirement age) who had either retired or, if they continued to work, treated their professional activity as a passion or a way to earn additional income. Usually, these people were distanced from the problems arising from work, because they were aware of their own limitations and the inevitable approach of the end of life.

### 2.5. Statistical Analysis

Descriptive statistics were used to describe the collected data, and the numbers and percentages were also calculated. In the statistical analysis, statistical hypotheses were verified with the Kruskal–Wallis test, which was employed for comparing more than 2 independent groups. In our study, a nonparametric test was also used due to the lack of normal distribution in the compared groups (*p*-value for the Shapiro–Wilk test <0.05). The study results were compiled using the “STATISTICA 13.3” software and Microsoft Excel 2016. The significance of differences and relationships was found at a *p*-value < 0.05.

## 3. Results

The mean overall score of the significance of hope in the semantic space in the cognitive aspect of men dying of cancer was 5.13 ± 1.42. This means that the respondents had a much more affirming perception of hope, although they were inclined to negate it to some extent. A definitely positive (*n* = 38; 34.55%) and positive (*n* = 36; 32.73%) reference to hope in the cognitive aspect was found in 74 out of 110 men in the terminal stage of cancer (in total). The respondents least often described hope as definitely pejorative (*n* = 3; 2.73%) and negative, i.e., pejorative (*n* = 5; 4.55%). A total of 92 out of 110 surveyed men (83.6%) had an affirmative reference to hope in the cognitive aspect. Detailed results of the conducted research concerning the perception of the importance of hope in the cognitive aspect presented by men in the terminal stage of cancer are presented in Table 2.

Specifying hope in the cognitive aspect, the surveyed men much more often indicated those that expressed affirmation rather than negation of hope within the individual pairs of semantically contradictory adjectives. Detailed data concerning this issue are presented in Table 3.

### 3.1. Hope True—False in the Semantic Space

The vast majority of the surveyed men (*n* = 75; 69.18%) indicated that hope was, to some extent, true for them. Indeed, for 35 (31.82%) of these individuals, it was definitely true, for 23 (20.91%) it was true and for 17 (15.45%) it was rather true. However, for 20 (18.18%) of the surveyed, hope was false—although the degree of its negation varied. For eight (7.27%) of them, hope was definitely false, according to seven (6.36%) it was false and according to five (4.55%) it was rather false. Of note, 15 (13.64%) of the surveyed men expressed indecision or lack of opinion whether their hope was, to some extent, false or true. These results are summarized in Table 4.

### 3.2. Hope Wise—Stupid in the Semantic Space

The vast majority of the surveyed men (*n* = 73; 66.36%) indicated that their hope was, to some extent, characterized by wisdom. For 29 out of 110 (26.36%) of them, it was definitely wise, for 29 (26.36%) it was wise and for 15 (13.64%) it was rather wise. For 19 (17.27%) of the surveyed men, hope was stupid, although the reference to it was not uniform. For nine (8.18%) of them, hope was definitely stupid, according to two (1.82%) it was stupid and according to eight (7.27%) it was rather stupid. Beyond the aforementioned, 18 (16.36%) of the surveyed men expressed indecision or a lack of opinion whether their hope was to some extent wise or stupid. These results are summarized in Table 5.

### 3.3. Hope Meaningful—Meaningless in the Semantic Space

The vast majority of the surveyed men (*n* = 81; 73.63%) indicated that hope made some sense to them. For 33 out of 110 (30.0%), hope was definitely sensible, for 29 (26.36%) it made sense and for 19 (17.27%) it was rather reasonable. For 15 (13.64%) of the surveyed, hope was pointless, although the reference to its pointlessness varied to some extent. For five (4.55%) of them, hope was definitely meaningless, for three (2.73%) it was meaningless and for seven (6.36%) it was rather meaningless. Indecision or a lack of opinion as to whether hope was to some extent sensible or nonsensical was expressed by 14 (12.73%) of the surveyed men. These results are summarized in Table 6.

### 3.4. Hope Real—Deceptive in the Semantic Space

The vast majority of the surveyed men (*n* = 64; 58.19%) indicated that hope was, to some extent, real for them. For 36 (32.73%), it was definitely real, for 15 (13.64%) it was real and for 13 (11.82%) it was rather real. For 27 (24.55%) of the surveyed men, hope was deceptive, although the reference to its illusory nature was varied to an average degree. For five (4.55%) of them, hope was definitely deceptive, for three (2.73%) it was deceptive and for seven (6.36%) it was rather deceptive. Of interest, as many as 19 (17.27%) of the surveyed men expressed indecision or a lack of an opinion whether their hope was to some extent deceptive or real. These results are summarized in Table 7.

### 3.5. Hope in the Semantic Space Defined Cognitively as Expressed by Men Dying of Cancer in Relation to the Age Group

Regarding the cognitive aspect of hope, the highest scores were obtained for men up to 35 years of age, while these were slightly lower among men from 36 to 55 years. The lowest results of the perception of hope in the cognitive aspect were observed in the age groups of 56–65 years and over 65 years of age.

The statistical analysis revealed no significant differences in the perception of hope in the cognitive aspect, depending on the age group of the surveyed men. A detailed analysis of the data shows that the average result for three age groups (18–35, 36–55 and over 65 years) indicates a positive perception of hope in the semantic space in the cognitive aspect by men in the terminal stage of cancer. The statistical analysis of the relationship between the perception of hope in the cognitive aspect and the age of the respondents is presented in Table 8.

When analyzing the results concerning the cognitive aspect of hope in the age group 18–35 years, it can be noticed that they were clearly shifted towards its affirmation. The average score for perceiving hope in the cognitive aspect was 5.46 ± 1.35, indicating a positive perception of it. However, it should be noted that the lowest result obtained was 3.0, which indicates a rather negative perception of hope.

The surveyed men belonging to the second age group (36–50 years) obtained a mean of 5.36 ± 1.27 in the cognitive aspect, which demonstrates that they perceived hope in a positive way. Mean plus one standard deviation shows that some perceived hope even more positively, while mean minus one standard deviation indicates that some respondents did not perceive it either positively or negatively.

In the age group of 51–65 years, the average score for the cognitive aspect of hope was 4.88 ± 1.44, which indicates that they perceived hope neither positively nor negatively, i.e., most of them found it difficult to define their attitude to it. It is worth noting that mean plus one standard deviation shows a positive and definitely positive attitude of the respondents to hope. Mean minus one standard deviation demonstrates that the respondents perceived hope neither positively nor negatively, although with a tendency towards a negative attitude to it.

The average cognitive result of perceiving hope among the surveyed men aged over 65 was 5.13 ± 1.53. This means that their perception of hope is positive. Mean plus one standard deviation shows an even more positive perception of hope in this aspect. Mean minus one standard deviation shows that they find it difficult to specify whether hope is positive or negative for them, with a tendency towards a rather negative attitude.

## 4. Discussion

The presented research on hope focuses on the cognitive connotations of the concept of hope in men who are confronted with their own death because they are in the terminal phase of cancer. In this mental state, they should not hope to be cured. However, they may have different references to hope, which in their case may be judged as “mother of fools”, fraudulent, senseless or false. The research shows that every patient in the terminal phase of cancer experiences his/her hope in a very personal and unique way. This fact was emphasized by the American doctor of Swiss origin Elisabeth Kübler-Ross as part of her psychological theory of the patient’s reaction to the news of an incurable disease and the imminent prospect of death [28].

Both the will to live and the expectation of a “miracle” by patients in the terminal phase of cancer may lead to the use of hope as a defense mechanism and/or a mechanism for coping with stress in such a difficult existential situation as facing death. In a situation where the dying person is left alone and does not experience the closeness of people important to him, he or she often loses all hope or concentrates with all his/her strength on an unrealistic hope for further life [30].

Hope, that is the desire of a given person for a certain positive and expected state of affairs, may have a therapeutic meaning for that individual. Hope that is not stupid, false, deceptive and meaningless can significantly affect the quality of life of patients in the terminal stage of cancer and help them come to terms with the inevitability of death. Thanks to wise, true, real and meaningful hope, patients in the terminal stage of neoplastic disease have better adaptability to the situation in which they find themselves. These patients make better use of their coping skills in dealing with the overwhelming suffering and the paralyzing fear of impending death. This has a positive impact on their well-being and the level of quality of life. In contrast, stupid, false, deceptive and meaningless hope, which has a destructive effect, because in its essence it is hopelessness/despair, contribute to the exacerbation of depression, as well as the intensification of physical symptoms of the disease, which may even strengthen the desire to hasten death.

Characterizing hope in the cognitive aspect of patients with terminal cancer may be helpful in developing an interdisciplinary bio–psycho–social–spiritual care strategy. Understanding and analyzing the causes that decrease stress levels and increase the level of positively directed hope will allow one to plan integral care. Consequently, the undertaken medical interventions will alleviate the symptoms characteristic of the terminal stage of cancer and better prepare the patient approaching the end of his/her life [31].

The conducted research was aimed at characterizing the degree of affirmation of hope of men dying of cancer in the cognitive aspect (using four pairs of semantically contradictory adjectives). The analysis of the literature on the subject [32,33,34] demonstrates that cancer patients who express hope positively and adequately to their health condition have a greater sense of self-agency, more often correctly use adaptive strategies of coping with stress resulting from the disease and are characterized by a lower level of various types of negative feelings. It is commonly known that hope gives meaning to human life, as well as stimulates the desire to fight for health, even in those situations in which, from a medical point of view, there is no biological viability to live or when all available treatment options to restore the patient’s health have already been used. Healthy hope (that is, wise, true, real and meaningful) in the terminal stage of cancer may be helpful in achieving the highest possible quality of life for the patient in the various stages of dying. Hence, at the end of their lives, these sick people will not despair but will accept the inevitability of their impending death. The analysis of the cognitive aspect of the semantic space of hope enables deepening patients’ existential reflection on themselves and their health situation, and this may significantly contribute to a more effective use of the mechanisms of coping with the terminal illness and acceptance of the inevitability of death.

So far, research devoted to the hope of people with cancer has been conducted in Poland by Krystyna de Walden-Gałuszko [8,22,23], Bogusław Block [35,36,37,38,39], A. Pietrzyk and S. Lizińczyk [40], as well as J. Trzebiński and M. Zięba [41]. However, our research on hope in the semantic space measured with the use of the semantic differential is a pioneering study. The analysis of relevant literature revealed no studies related to this issue in the databases. It is, therefore, advisable to undertake more comprehensive research concerning hope, both cognitively and in other aspects (e.g., functional and emotional). It may be assumed that the perception of hope in the semantic space of the cognitive aspect by patients in the terminal stage of cancer affects their life attitudes, as well as their emotional and social functioning. Hence, they have a more qualitative relationship with themselves and others, and they are also better able to cope with the stress of suffering and imminent death. Defining hope in the cognitive aspect, the surveyed men more often affirmed than negated it. According to them, hope was more true, wise, meaningful and real than false, stupid, meaningless or deceptive. Among the adjectives that define hope in cognitive terms, the most varied results concerned hope in relation to the pair of adjectives deceptive–real. This probably resulted from the awareness of the respondents that there was no chance of recovery and their lives would end shortly. A positive perception of hope in the cognitive aspect may be associated with positive experiences related to hope earlier in patients’ lives, so it is likely that hope was more positively defined by people who had never been disappointed in the experienced personal hope in relation to prior life situations. In all expressions of hope in the cognitive aspect, most of the respondents affirmed hope.

It is worth noting that 18 men denied hope when choosing concepts that denied hope, which indicates that they were cognitively overwhelmed by hopelessness as regards their existential experience. Although usually hopelessness/despair is perceived as an emotional state, the cognitive sphere is its important component. The respondents declared their attitude to hope by selecting one of seven possibilities (from definitely false/stupid/meaningless/deceptive to definitely true/wise/meaningful/real). The results of the research were varied. The respondents, based on their own experience and convictions, selected the degree of specifying hope within the pairs of adjectives offered to them. The researchers also wondered whether there were differences in the perception of hope in the semantic space in the cognitive area due to the respondents’ belonging to different age groups but the same stage of cancer (i.e., terminal phase). However, statistical analysis did not reveal any significant differences in the general perception of hope depending on the age of the surveyed men.

The cognitive aspect of hope is very important for men in the terminal phase of cancer, because it has a large impact on the positive stimulation of realistic hope in all spheres of their existence. Thanks to this, they go through the stages of dying more easily and are better prepared for imminent death. The analysis of the collected data is of universal importance, because hope is inscribed in the structure of human life as an important motivating force to struggle with everyday existential problems, especially in extreme situations.

### Study Limitations

The criterion for selection in the research was the age of 18. The patient had to agree to participate in the study. Some patients refused to participate in the study because they felt too weak. Some patients did not complete the study due to the sudden deterioration in health.

## 5. Conclusions

The surveyed men in the terminal stage of cancer most often perceived hope in the semantic space in the cognitive aspect as more true, wise, meaningful and real than false, stupid, meaningless and deceptive. Their attitude to hope was, therefore, more affirmative than negative.

The research did not reveal the importance of the age of the respondents on the degree of affirmation/negation of hope in the cognitive aspect in the semantic space.

Men in the period of late maturity and professional activity expressed the lowest level of the affirmation of hope.

It is worthwhile to conduct further research concerning hope in other aspects (especially emotional and functional) in the semantic space in order to use the obtained results to consider what to take into account when providing patients in the terminal stage of cancer with better personalized holistic care than before.

## Figures and Tables

**Table 1:** Characteristics of the studied group.

Characteristics of the Studied Group (*n* = 110)			
Variable		M ± SD	Me (Min., Max.)
Age		60.15 ± 13.53	60.0 (19.0, 90.0)
Duration of the disease (months)		35.84 ± 56.96	18.0 (1.0, 480.0)
		*n*	%
Place of residence	village	30	27.27
	city up to 50 thousands inhabitants	27	24.55
	city 50–100 thousands inhabitants	12	10.91
	city over 100 thousands inhabitants	41	37.27
Housing	on one’s own	26	23.64
	with family	77	70.0
	other	7	6.36
Marital status	single	17	15.45
	married	58	52.73
	widowed	27	24.55
	divorced	8	7.27
Education	elementary	17	15.45
	vocational	28	25.45
	secondary	36	32.73
	BA or equivalent	12	10.91
	MA or equivalent	17	15.45
Material status	bad	25	22.73
	satisfactory	51	46.36
	good	31	28.18
	very good	3	2.73
Type of cancer	upper respiratory tract	7	6.36
	respiratory system	13	11.82
	nervous system	8	7.27
	digestive system	14	12.73
	urinary system	3	2.73
	osteoarticular system	6	5.45
	endocrine system	3	2.73
	hematopoietic system	3	2.73
	sexual system	40	36.36
	skin	1	0.91
	other	12	10.91
Type of institution providing assistance	stationary hospice	51	46.36
	home hospice	53	48.18
	palliative medicine ward	6	5.46

**Table 2:** The degree of affirmation/negation of hope in the cognitive aspect presented by men in the terminal stage of cancer (*n* = 110).

Degree of Affirmation/Negation of Hope	*n*	%
Definitely positive	38	34.55
Positive	36	32.73
Rather positive	18	16.36
Rather negative	10	9.08
Negative	5	4.55
Definitely negative	3	2.73
Total	110	100%

**Table 3:** Perception of the meaning of hope in the cognitive aspect by the surveyed men in the semantic space measured with the DSN-3 test—descriptive statistics.

Adjective Pairs That Define Hope in the Cognitive Aspect	*n*	M	SD	Min.	Max.	Me
False–true	110	5.70	1.88	1.0	7.0	6.00
Stupid–wise	110	5.10	1.81	1.0	7.0	6.00
Meaningless–meaningful	110	5.35	1.65	1.0	7.0	6.00
Deceptive–real	110	4.93	1.93	1.0	7.0	5.00
Overall Score	110	5.13	1.82	1.0	7.0	5.75

**Table 4:** The degree of affirmation/negation of hope in the cognitive aspect in relation to the pair of adjectives false–true expressed by men in the terminal stage of cancer (*n* = 110).

The Degree of Affirmation/Negation of Hope in the Cognitive Aspect in Relation to the Pair of Adjectives False–True Number of Responses *n* (%)						
Definitely False	False	Rather False	Neither False nor True (I Have No Opinion)	Rather True	True	Definitely True
8 (7.7%)	7 (6.36%)	5 (4.55%)	15 (13.64%)	17 (15.45%)	23 (20.91%)	35 (31.82%)

**Table 5:** The degree of affirmation/negation of hope in the cognitive aspect in relation to the pair of adjectives stupid–wise expressed by men in the terminal stage of cancer (*n* = 110).

The Degree of Affirmation/Negation of Hope in the Cognitive Aspect in Relation to the Pair of Adjectives Stupid–Wise Number of Responses *n* (%)						
Definitely Stupid	Stupid	Rather Stupid	Neither Stupid nor Wise (I Have No Opinion)	Rather Wise	Wise	Definitely Wise
9 (8.18%)	2 (1.2%)	8 (7.27%)	18 (16.36%)	15 (13.64%)	29 (26.36%)	29 (26.36%)

**Table 6:** The degree of affirmation/negation of hope in the cognitive aspect in relation to the pair of adjectives meaningless–meaningful expressed by men in the terminal stage of cancer (*n* = 110).

The Degree of Affirmation/Negation of Hope in the Cognitive Aspect in Relation to the Pair of Adjectives Meaningless–Meaningful Number of Responses *n* (%)						
Definitely Meaningless	Meaningless	Rather Meaningless	Neither Meaningless nor Meaningful (I Have No Opinion)	Rather Meaningful	Meaningful	Definitely Meaningful
5 (4.55%)	3 (2.73%)	7 (6.36%)	14 (12.73%)	19 (17.27%)	29 (26.36%)	33 (30.0%)

**Table 7:** The degree of affirmation/negation of hope in the cognitive aspect in relation to the pair of adjectives deceptive–real expressed by men in the terminal stage of cancer (*n* = 110).

The Degree of Affirmation/Negation of Hope in the Cognitive Aspect in Relation to the Pair of Adjectives: Deceptive–Real Number of Responses *n* (%)						
Definitely Deceptive	Deceptive	Rather Deceptive	Neither Deceptive nor Real (I Have No Opinion)	Rather Real	Real	Definitely Real
5 (4.5%)	12 (10.91%)	10 (9.09%)	19 (17.27%)	13 (11.82%)	15 (13.64%)	36 (32.73%)

**Table 8:** Perception of hope in the cognitive aspect depending on the age of the surveyed men—measured via the DSN-3 test.

Age	*n*	Cognitive Aspect of Hope					Kruskal–Wallis Test	
		M	SD	Min.	Max.	Me	H	*p*
18–35 years	6	5.46	1.35	3.0	6.75	5.88	2.00643	0.5711
36–55 years	29	5.36	1.27	2.25	7.0	5.50		
56–65 years	35	4.88	1.44	1.0	7.0	5.50		
Over 65 years	40	5.13	1.53	1.0	7.0	5.50		
Overall	110	5.13	1.42	1.0	7.0	5.50		

## Data Availability

The data presented in this study are available upon request from the corresponding author.
